# Identification of the Causal Agent of Aqueous Spot Disease of Sweet Cherries (*Prunus avium* L.) from the Jerte Valley (Cáceres, Spain)

**DOI:** 10.3390/foods10102281

**Published:** 2021-09-26

**Authors:** Manuel Joaquín Serradilla, Carlos Moraga, Santiago Ruiz-Moyano, Paula Tejero, María de Guía Córdoba, Alberto Martín, Alejandro Hernández

**Affiliations:** 1 Instituto Tecnológico Agroalimentario de Extremadura (INTAEX), Centro de Investigaciones Científicas y Tecnológicas de Extremadura (CICYTEX), Avd. Adolfo Suárez s/n, 06007 Badajoz, Spain; manuel.serradilla@juntaex.es; 2 Nutrición y Bromatología, Escuela de Ingenierías Agrarias, Universidad de Extremadura, Avd. Adolfo Suárez s/n, 06007 Badajoz, Spain; cmoragaloz@gmail.com (C.M.); patejeroc@gmail.com (P.T.); mdeguia@unex.es (M.d.G.C.); amartin@unex.es (A.M.); ahernandez@unex.es (A.H.); 3 Instituto Universitario de Investigación en Recursos Agrarios (INURA), Universidad de Extremadura, Avd. de la Investigación s/n, 06006 Badajoz, Spain

**Keywords:** emerging disease, preharvest, postharvest, Enterobacterales, latent infection, *Botrytis cinerea*

## Abstract

The pre and postharvest disease named ‘aqueous spot’ is an emerging risk for sweet cherries growing in Jerte Valley (Cáceres, Spain). Early stages of the disease appear in the tree, but it is usually detected after harvesting, during the postharvest period. Symptoms include the appearance of skin discolouration and translucency in the shoulder areas. At the most advanced stages, a mycelium of white colour partially or completely covers the fruit. This manuscript provides a detailed description of the microbes involved in this disease, such as bacteria, yeasts, and moulds. Microbes of different cherry cultivars were studied during two consecutive seasons (2019 and 2020). The counts of bacteria and yeast in damaged tissues were higher (7.05 and 6.38 log10 CFU/g for total aerobic mesophilic microbes and yeasts, respectively) than sound tissues (6.08 and 5.19 log10 CFU/g, respectively). The Enterobacterales order dominated the bacteria population. Among yeasts, *Yarrowia lipolytica*, in 2019, and *Metschnikowia pulcherrima* and *Metschnikowia viticola*, in 2020, were consistently isolated from all samples. The presence of moulds was inconsistently detected at the early stage of this disease by plate counts. However, microscopic observations revealed the presence of hyphae in cherry flesh. Different pathogenic moulds were identified, although white mycelium, identified as *Botrytis cinerea* by molecular methods, was consistently isolated at later stages. Inoculation tests confirmed the involvement of white-mycelium *B. cinerea* in the development of this new postharvest disease in the Jerte Valley. Its combination with Enterobacterales enhanced the evolution of rotting, whereas the combination with yeasts decreased and delayed the symptoms. This work presents the first report of a consortia of microorganisms implicated in the development of ‘aqueous spot’, an emerging disease in sweet cherry cultivars in the Jerte Valley.

## 1. Introduction

The Jerte Valley (Extremadura, Southwest of Spain) is the most important sweet cherry (*Prunus avium* L.) production area in Spain. This region has an area of 7524 ha, with a potential yield of more than 41,000 tons [1]. The production is mainly exported to European countries, such as the United Kingdom, Germany, France, and Italy. Besides the autochthonous ‘Picota’ type cherries [2,3,4], the catalogue of cultivars in the Jerte Valley is extensive, producing from the end of April to the beginning of August. Among the early cultivars, the most important regarding yield is ‘Burlat’, ‘Van’ as a mid-season cultivar, and finally, as late cultivars, ‘Lapins’ and ‘Sweetheart’.

Sweet cherries are highly perishable because of their high respiration rate, and they can be damaged by mechanical forces, physiological disorders, or microbiological spoilage. Surface pitting caused by impact damage is the major mechanical postharvest deterioration [5,6]. Moulds are the primary cause of microbial damage. The occurrence of brown rot in sweet cherries has been widely documented and can be caused by *Monilia fructicola* [7], *Monilia fructigena* [8], and *Monilia laxa* [9]. Different species of *Penicillium* have been responsible for cherry spoilage, such as *Penicillium expansum* [10], *Penicillium crustosum* [11], and *Penicillium oxalicum* [12]. *Botrytis cinerea* [13,14], *Alternaria alternata* [15], and *Rhizopus stolonifer* [16] are the other pathogens reported as rot causing agents in sweet cherries. Recently, *Mucor piriformis* A. Fisch has been linked to a postharvest disease called cherry ‘slip-skin’ and is characterised by its occurrence mainly in late cultivars, such as ‘Lapins’ and ‘Stacatto’. This disease is characterised by a softening of shoulders due to maceration of the subepidermal tissue that causes the skin to slip off easily [17,18].

As far as we know, there are no reports about yeast spoilage on sweet cherries in the literature, except for Kim [19] who reported the first case of postharvest cherry rot caused by *Aureobasidium pullulans*. Apart from that, the occurrence of sour rot in stone fruit has increased in the last years. *Geotrichum candidum*, *Issatchenkia scutulata*, and *Kloeckera apiculata* were consistently isolated in sour, rotten peaches and nectarines from California [20]. Another study reported *G. candidum* in sour stone fruit [21]. As with yeasts, reports about bacteria involvement in cherry spoilage are limited. Some reports associated *Pseudomonas syringae* with bacterial canker in cherry trees [22,23], although its postharvest effect remains unclear.

**Figure 1:**
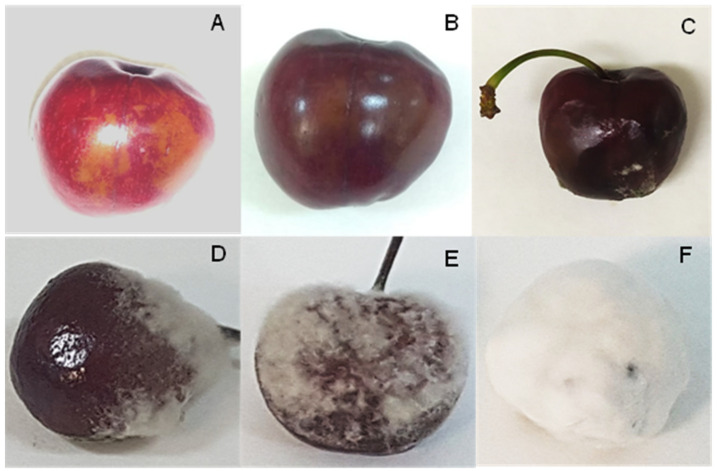
Evolution of the symptomatology of ‘aqueous spot’ disease. (**A**,**B**) partial decolouration and translucent cherry skin; (**C**) softening of tissues, initial development of white/grey mycelium; (**D**,**E**) development of white mycelium; (**F**) complete coverage of a cherry by white mycelium.

Beyond the abovementioned diseases, a new pathology is emerging in cherry production in the Jerte Valley denominated by the industry of cherry as ‘aqueous spot’. The first finding of the emerging ‘aqueous spot’ in the sweet cherries from the Jerte Valley is not clear. However, in the last four years, its occurrence has grown dramatically, affecting mostly early cultivars, with ‘Burlat’ having the highest occurrence with 90% of total yield. In addition, cultivars such as ‘Sweetheart’ and ‘Lapins’, which ripen late, are also beginning to be affected. Preliminary observations indicate that its occurrence becomes more acute after periods of rainfall with temperature increases. The causal agent of the denominated ‘aqueous spot’ is still unclear, and both physiological and microbiological origins have been postulated. The symptomatology of ‘aqueous spot’ at an early stage is partial decolouration and translucency under the skin. This disease becomes more acute during subsequent postharvest life by increasing the area of damage, and softening and darkening the tissue, so that skin slippage can occur after touching it. In the last stage, white or grey mycelium appears in the affected area. Finally, the cherry is completely covered by a white mycelium (Figure 1). Early symptoms usually appear on the tree before harvest, but it progresses throughout postharvest life as mentioned above. To the best of our knowledge, scientific information on this symptomatology in cherries from Jerte Valley (Cáceres, Spain) has not been addressed; thus, the disease’s origin remains unknown. Recently, Spadoni et al. [24] reported a similar disease on sweet cherries from Bari (Italy), identifying the causal agent as *Stemphylium eturmiunum*. Previously, O’Gorman et al. [18] reported another disease named ‘slip-skin’ whose causal agent is *Mucor piriformis* A. Fisch.

The productive cherry sector in the Jerte Valley needs to control the emerging disease called ‘aqueous spot’. It is important to determine the possible origin and factors that favour its occurrence. Thus, the aim of this work was to elucidate the causal agent of this pathology. For that, an extensive analysis of the microbial population present during the development of ‘aqueous spot’ was performed. Finally, inoculation trials confirmed the specific identification of the microbes involved in this disease.

## 2. Materials and Methods

### 2.1. Sample Collection

All cherry samples, at commercial maturity stage 4 according Serradilla et al. [25], were provided by the ‘Agrupación de Cooperativas del Valle del Jerte SC.L.’ (https://www.ac-vallejerte.es/, accessed on 25 July 2021) during the 2019 and 2020 seasons. They were transported under refrigerated conditions (<5 °C) to the Agricultural Engineering School facilities from the University of Extremadura (Badajoz, Spain) and processed in less than 24 h. 

Eight batches of cherry samples with ‘aqueous spot’ symptoms were analysed in 2019. They belonged to the ‘Burlat’ (samples B1 and B2), ‘California’ (samples C1 to C4), and ‘Sweetheart’ (samples S1 and S2) cultivars. In 2020, three batches of samples belonging to the ‘Lapins’ (samples L1 and L2), and ‘California’ (sample C5) cultivars were analysed. The samples consisted of boxes with 1.5–5 kg of cherries. In addition, for microbial inoculation assays, undamaged cherries belonging to ‘Burlat’, ‘California’, and ‘Lapins’ cultivars were used.

### 2.2. Sample Preparation, Microbiological Counts and Isolation of Microbes

To elucidate the causal agent of ‘aqueous spot’ disease, 10 cherries with initial symptoms by batch were selected for microbial analysis. Initial symptoms were characterised by small translucent areas with a loss of firmness to the touch (Figure 1A,B). Damaged tissue and sound tissue of cherries were analysed separately. The damaged tissue area was aseptically separated with a scalpel, weighted, diluted 10 times with sterile peptone water and homogenized for 120 s in a Stomacher instrument (Lab-Blender 400, Seward, London, the UK). After homogenisation, decimal serial dilutions were performed with sterile peptone water. Finally, 0.1 mL was seeded on plate count agar (PCA, 30 °C/24 h) for total aerobic mesophilic microorganisms (TAM) counts and potato dextrose agar acidified to pH 3.5 with a sterilised solution of tartaric acid (PDA, 25 °C/5 days) for yeast and mould counts. The same procedure was performed with sound tissues. The microbial analysis was performed in triplicate by each batch.

In parallel, cherry samples were immersed in 100 mg/L sodium hypochlorite solution for 3 min to sanitise the surfaces of the cherries. Cherries were air-dried, and then microbiological analysis was carried out as above in damaged and sound cherry tissues. As a control, cherries without ‘aqueous spot’ symptoms were analysed. The analyses were performed in triplicate, and microbial counts were expressed as log_10_ CFU/g of cherries.

The isolation of microbes, bacteria and fungi was performed based on the morphology of colonies. Three colonies of each morphology detected from the most diluted plates were isolated by streaking in the specific culture media (PCA and PDA). Once pure colonies were obtained, cultures were stored at −80 °C in 40% sterile glycerol.

An alternative procedure for the isolation of moulds was performed. Three cherries by sample with ‘aqueous spot’ symptoms were individually stored in sterile plastic bottles at room temperature (~20–25 °C) until mycelia development was evident (Figure 1E,F). The mould was directly inoculated onto acidified PDA plates with a loop. After incubation at 25 °C/5 days, mould colonies were isolated as above. As controls, cherries without ‘aqueous spot’ symptoms were also stored in sterile plastic bottles at room temperature. 

### 2.3. Identification of Isolated Microbes

Genomic DNA from microbial isolates, bacteria, and fungi was extracted using the NucleoSpin^®^ Microbial DNA Kit (Macherey-Nagel, Düren, Germany) according to the manufacturer’s instructions. The DNA concentration and quality were assessed by a Nanodrop ND-1000 spectrophotometer (Thermo Fisher Scientific, Waltham, MA, USA). To identify the isolates at the species level, for bacteria, the 16S rRNA gene was amplified by PCR using the universal primers 27F and 1492R as described by Ruiz-Moyano et al. [26]. For fungi, the internal transcribed spacer ITS1/ITS2-5.8S rRNA region was amplified as detailed by Gallardo et al. [27] using the primer pairs ITS1 and ITS4 described by White et al. [28]. The resulting amplicons were visualised after electrophoresis on a 1% agarose gel stained with Midori Green Advance (Nippon, Tokyo, Japan). The Generuler 100 bp plus DNA ladder (Thermo-Fisher Scientific, San Jose, CA, USA) was used as the reference. The PCR products were purified using the Genejet PCR Purification Kit (Thermo-Fisher Scientific) and then sequenced at the Service of Bioscience Applied Techniques (STAB) of the University of Extremadura (Badajoz, Spain). The sequences were analysed and edited with Chromas Pro version 1.49 beta (Technelysium, City, QL, Australia) and checked by nucleotide–nucleotide comparison at EMBL/GenBank databases using the BLAST algorithm. The identities of the isolates were determined based on the highest score.

Additionally, identities of the yeast isolates identified by ITS sequencing as *Metschnikowia* spp. were confirmed by amplification of the D1/D2 domain of the 26S large ribosomal subunit (LSU) of rRNA using the primers NL1 and NL4 [29], following the PCR conditions described by Gallardo et al. [27] and subsequent sequencing of the PCR amplicons as above.

### 2.4. Microscopic Observation of Cherry Tissues

Cherries with initial ‘aqueous spot’ symptoms (Figure 1A,B) from all batches were selected for tissue observation. The damaged portion was aseptically obtained and smoothly homogenised. After that, direct observations were performed. Microscopic observations were made at 40× magnification with sub-stage illumination (DMLS, Leica, Buccinasco, MI, Italy). The structures were visualised with a Leica DM 2000 LED microscope. 

### 2.5. Inoculation Assays

The effect of microbes isolated from samples with symptoms of disease (Section 2.2) on cherries was assessed by direct inoculation. Undamaged cherries of ‘Burlat’, ‘California’ and ‘Lapins’ cultivars were used. After sanitisation, cherries were injured with a sterile tip (one wound per cherry, ~3 mm of diameter × 3 mm of depth).

Pure cultures of bacteria were grown in nutrient broth at 30 °C for 24 h and recovered by centrifugation at 10,000× *g* for 3 min. Pellets were suspended in sterile water. Pure cultures of yeast were grown in acidified PDA at 25 °C for 48 h. A loop of yeast was suspended in 500 μL of sterile water. Pure mould cultures were grown in acidified PDA at 25 °C for 10 days. Conidia was recovered with a sterile 0.05% solution of tween 80. Microbe solutions were quantified by direct observation using an optical microscope in a Neubauer chamber. As inoculation suspensions, bacteria and yeasts were diluted to 10^7^ cells/mL, and conidia was diluted to 10^5^ spores/mL. 

Ten injured cherries were inoculated with 5 μL of each microbe solution for the individual inoculations. Each cherry was stored individually in a sterile plastic bottle at 25 °C for 10 days. 

A selection of bacteria, yeasts and moulds was made based on their frequency of isolation, incidence and symptoms in the assays mentioned above for combinations of double and triple inoculations. Then, combined inocula were carried out with mould × bacteria, mould × yeast and finally mould × bacteria × yeast. 

In all inoculation tests, the incidence percentages and rotting symptoms were recorded daily, and pictures were taken periodically. The assays were performed in triplicate. Percentages of incidence were converted into Bliss angular values before analysis. 

To know the capacity of the potential agents to infect sound sweet cherries, an additional inoculation assay was carried out by immersion in the selected moulds. To do this, 10 cherries, free of defects, were immersed for 3 min in a solution of conidia at a concentration of 10^5^ spores/mL. After air-drying in an aseptic environment, cherries were placed in sterile plastic containers and incubated at 25 °C for five days. The assays were performed in triplicate.

### 2.6. Genetic Characterisation of Botrytis Cinerea Morphotypes 

To characterise *Botrytis cinerea* isolates, two representative isolates from the four morphotypes detected in inoculation assays (Section 2.5) were selected. A multi-locus approach using four phylogenetic markers was used. These were partial fragments of ITS, LSU 26S rDNA, calmodulin and β-tubulin regions. The PCR amplification of the ITS region was carried out using the primers ITS1 and ITS4 [28], LSU 26S rDNA using the primers NL1 and NL4 [29], calmodulin using primers CMD5 and CMD6 [30], and β-tubulin using primers Bet2a and Bet2b [31]. PCR amplification was performed in 50 μL of reaction mixtures containing 10 ng of genomic DNA, 50 pmol of the forward primer and reverse primer, 0.2 mM of each dNTP, 0.1 vol of 10X PCR buffer, and 1.25 U Green DreamTaq DNA polymerase (Fermentas, Thermo Fisher Scientific Inc., Waltham, MA, USA). PCR reactions were run in a T100™ Thermal Cycler (BioRad, Hercules, CA, USA) using an initial denaturation at 94 °C for 4 min, followed by 35 cycles of 94 °C for 1 min, annealing at 55 °C for 1 min, elongation at 72 °C for 1 min, and a final extension at 72 °C for 10 min. PCR amplicons were visualised and sequenced as above. 

A phylogenetic tree was inferred by using the maximum likelihood method and the Tamura–Nei model [32] constructed with the concatenated sequences of ITS1-5.8S rDAN-ITS4 + LSU 26S rDNA + calmodulin gene + β-tubulin of *Botrytis cinerea* morphotypes and type strains (CECT2850, CECT20516, CECT20518, and CECT20793). Evolutionary analyses were conducted using MEGAX [33]. 

In addition, inter simple sequence repeat (ISSR) PCR analyses were carried out using three selected primers, (GTG)_5_ (5′-GTGGTGGTGGTGGTG-3′), (CAG)_4_ (5′-ARRTYCAGCAGCAGCAG-3′), and UBC-809 (5′-AGAGAGAGAGAGAGAGG-3′), and following the PCR conditions described by Mahmoud et al. [34]. Amplification products were separated on a 2% agarose gel stained with Midori Green Advance (Nippon, Tokyo, Japan). The Generuler 100 bp plus DNA ladder (Thermo-Fisher Scientific, San Jose, CA, USA) was used as the reference.

Finally, molecular detection of the transposable elements *Boty* and *Flipper* was conducted as described in Muñoz et al. [35].

### 2.7. Analysis of Results

Data were evaluated statistically using SPSS for Windows (version 19.0; SPSS Inc., Chicago, IL, USA). Mean values of microbial counts were compared by one-way analysis of variance (ANOVA). Multiple comparisons of the means were performed using Tukey’s honestly significant difference (HSD) test (*p* ≤ 0.050). 

## 3. Results

### 3.1. Microbial Counts

**Figure 2:**
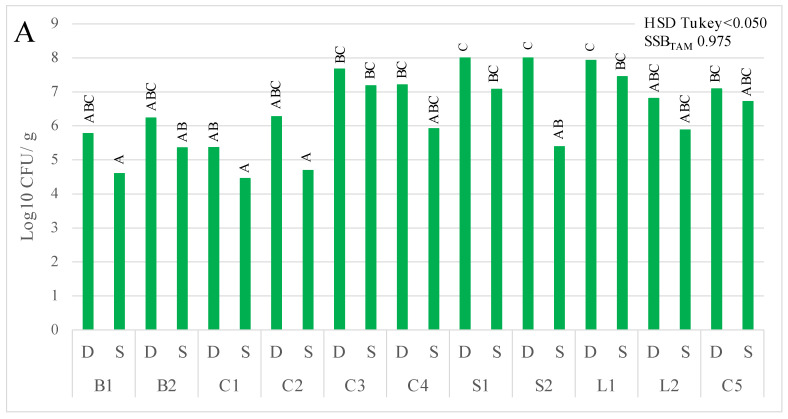

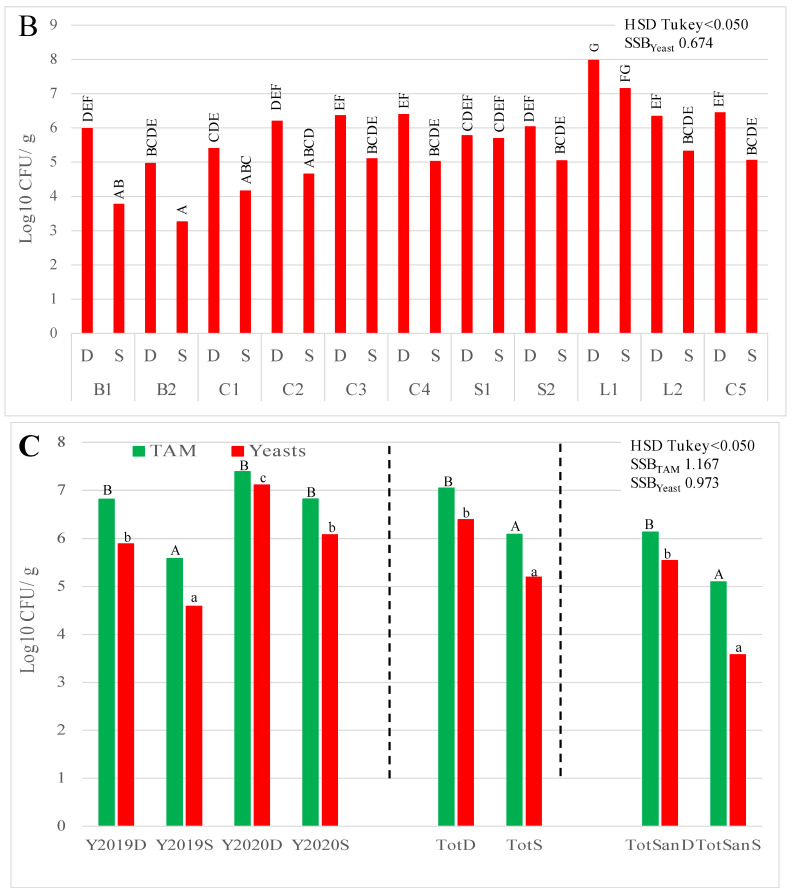
Counts of total aerobic mesophilic (TAM); (**A**) microorganisms and yeast (**B**) in damaged (D) and sound tissue (S) from (**A**) samples of cherries in 2019 (B1, B2, C1–C4, S1, S2) and 2020 (L1, L2, C5), and (**C**) mean counts by seasons: total counts by type of tissue (TotD: total counts on damaged tissues and TotS: total counts on sound tissues) and total counts by type of tissue after sanitisation with sodium hypochlorite (TotSanD: total counts on sanitized damaged tissues and TotSanS: total counts on sanitized sound tissues). Bars with different upper-case letters for TAM and lower-case letter for yeast indicates statistical differences (*p* < 0.050). B, C, S, and L represents ‘Burlat’, ‘California’, ‘Sweetheart’, and ‘Lapins’ sweet cherry cultivars, respectively. HSD Tukey: Honestly Significant Difference; SSB: Statistical Significance Bar.

TAM and yeast counts of damaged (D) and sound (S) cherry tissues are shown in Figure 2. Damaged tissues showed TAM counts ranging from 5.37 ± 0.16 (C1) to 8.02 ± 0.7 (S1) log10 CFU/g of cherries. Meanwhile, sound tissues varied from 4.45 ± 0.77 (L3) to 7.44 ± 0.24 (L1) log_10_ CFU/g (Figure 2A). On the other hand, yeast counts of damaged tissues ranged from 4.96 ± 0.37 (B2) to 7.97 ± 0.34 (L1) log_10_ CFU/g, whereas counts for sound tissues were from 3.25 ± 0.31 (B2) to 7.15 ± 0.60 (L1) log_10_ CFU/g of cherries (Figure 2B). 

The analyses of TAM and yeast counts per season showed no differences between damaged tissues in both years. Meanwhile, sound tissues from 2020, had significantly (*p* < 0.050) lower counts than samples from 2019. However, the analyses of total counts from both seasons showed higher counts (*p* < 0.001) in damaged tissues (7.05 ± 0.98 and 6.39 ± 0.92 log_10_ CFU/g of cherries for TAM and yeast, respectively) compared to sound tissues (6.09 ± 1.24 and 5.19 ± 1.22 log_10_ CFU/g of cherries for TAM and yeast, respectively). Sanitisation of cherry surfaces reduced TAM and yeast populations by around 1 and 2 log_10_ CFU/g, respectively, with higher counts (*p* < 0.050) for damaged tissues than sound tissues (Figure 2C). Figure S1 shows in detail the TAM and yeast counts after sanitisation in all cultivars collected in 2019 and 2020 seasons.

**Figure 3:**
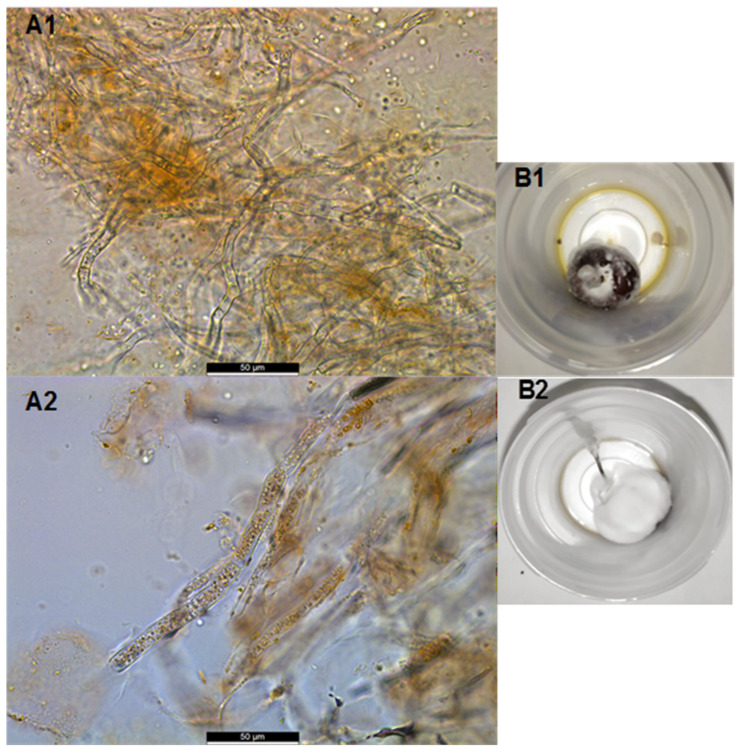
Fungal hyphae present in the flesh of ‘California’ (**A1**) and ‘Burlat’ (**A2**) cultivars (*Prunus avium* L.). Final symptoms of cherries affected by symptoms of ‘aqueous spot’, total coverage of white mycelium (**B1**) and grey mycelium (**B2**) after six days stored in sterile plastic bottles at 25 °C.

The presence of moulds was inconsistently detected on cherries. Mould colonies appeared on the least diluted plates, and media counts were always lower than the detection limit for microbial analyses (2 log_10_ CFU/g). However, microscopic observations of damaged tissues from all samples showed the presence of mycelium within the flesh, as shown in Figure 3A. Additionally, the final stage of this disease in the affected cherries inside the plastic bottles included complete coverage of the fruit with white, occasionally grey, mycelium (Figure 3B). 

### 3.2. Species Identification of Bacteria, Yeast, and Moulds

The bacteria identified on the cherries were dominated by species belonging to the Enterobacterales order, isolated in all cases and on both samples and seasons. *Rahnella aquatilis*, *Pantoea agglomerans*, *Tatumella tyseos*, *Hafnia paralvei*, *Rosenbergiella nectarea*, *Erwinia* spp. and *Lonsdalea quercina* were the species identified (Table 1). Regarding *Erwinia* spp., a more specific identification was not possible because BLAST gave a similar percentage of identities with different accession numbers in GenBank of non-pathogenic species such as *E. billingiae*, *E. tasmaniensis*, and *E. persicina*. *Bacillus thuringiensis* and *Agrococcus lahaulensis* were also identified in 2019, while *Leuconostoc mesenteroides* and *Pseudomonas* spp. were identified in 2020. All Enterobacterales species were found in all samples tested, both damaged and sound tissues, sanitised or not, as well as cherries without any disease symptoms.

Twelve yeast species were identified in cherry samples affected by ‘aqueous spot’ (Table 1). Candida oleophila, Candida railesensis, Aureobasidium pullulans, Filobasidium wieringae, Filobasidium magnum, Pichia kluyveri, and Hanseniaspora uvarum were found in both seasons, although they were not present in all samples. Yarrowia lypolitica, isolated from all samples, and Rhodotorula nothofagi were found in 2019; while Metschnikowia pulcherrima and Metschnikowia viticola, isolated from all samples, were found in 2020. The identification percentage of Metschnikowia species of ITS1-5.8S rDNA-ITS4 was below 97%, due to hairpins in the genomic ribosomal sequences [36]. The identification was confirmed by sequencing the 26S rDNA large subunit, with the percentage of identities above 99% (Table 1). Eleven out to twelve species were isolated from sound tissues. Geotrichum candidum was isolated in one 2020 sample from the lowest dilutions. The type of tissue (damaged or sound) and the hygienic conditions did not modify the identified species.

Mould presence was scarce in cherries with initial disease symptoms, as mentioned above. Eleven species were identified, although none were isolated from all samples. *Penicillium crustosum*, *Mucor plumbeus*, *Alternaria alternata*, *Cladosporium cladosporioides*, and *Botrytis cinerea* were isolated from samples in both seasons. *Schyzophylum commune* and *Trichodema atroviride* were found in samples from 2019, while *Fusarium oxysporum*, *Mucor racemosus*, *Monilia laxa*, and *Alternaria infectoria* were isolated from samples in 2020. 

When the disease advanced, cherries were completely covered by primarily white or grey mycelia. The isolation and identification of this mould consistently confirmed the presence of different morphologies of *B. cinerea* in the last stages of the disease. 

### 3.3. Inoculation of Bacteria, Yeast, and Moulds in Wounded Cherries

The results of the individual microbial inoculations of wounded cherries are shown in Table 1. When the rotting of cherries was observed, the inoculation of bacteria usually generated the browning of tissues and a slight liquefication around the wounds. *Erwinia* spp. produced bubbles inside of the wounds. Incidence percentages ranged from 0.0% (inoculations with *B. cereus* and *A. lahaulensis*) to 83.3% (*L. quercina*). 

**Figure 4:**
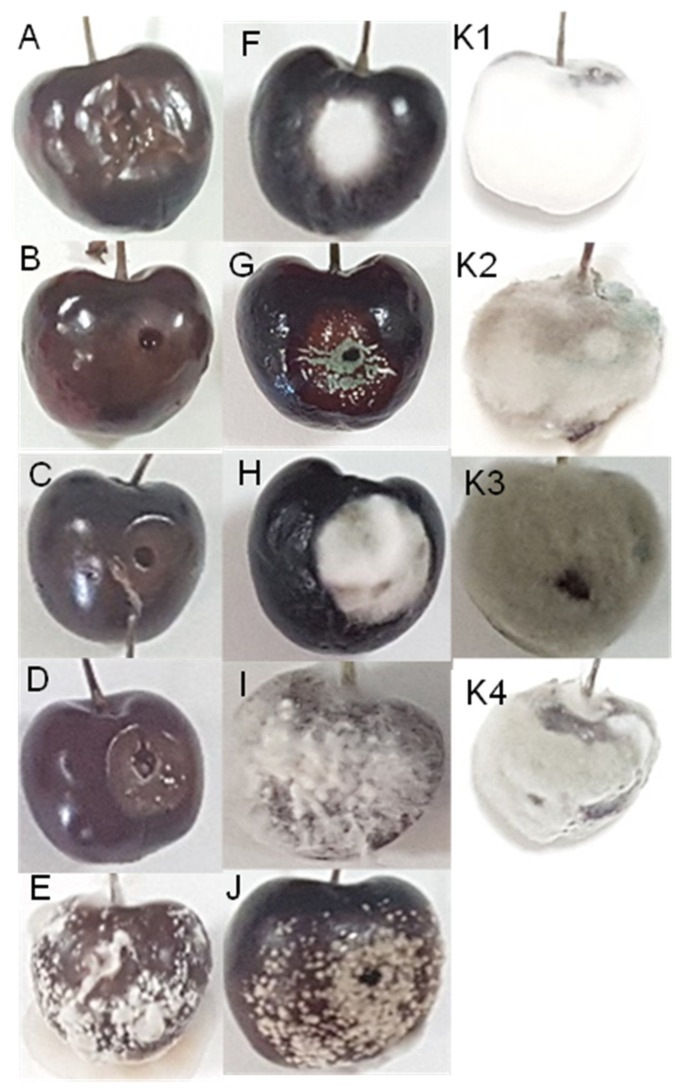
Cherries of the ‘Lapins’ cultivar inoculated with Lonsdalea quercina (**A**), Leuconoctoc mesenterioides (**B**), Hanseniaspora uvarum (**C**), Metschnikowia viticola (**D**), Geotrichum candidum (**E**), Fusarium oxysporum (**F**), Penicillium crustosum (**G**), Alternaria alternata (**H**), Schizophylum commune (**I**), Monilia laxa (**J**), and Botrytis cinerea isolates M2 (**K1**), M15 (**K2**), M31 (**K3**), and M44 (**K4**), stored at 25 °C for six days.

Yeast inoculation, in general, did not modify the tissue around wounds. *Hanseniaspora uvarum*, *M. pulcherrima* and *M. viticola* spoiled 13.3%, 20.0%, and 33.3% of cherries, respectively. The observed symptoms were mainly depression of wounds and slight browning of the tissues (Figure 4C,D). In contrast, *G. candidum* infected 100% of the inoculated wounds, with visual observation of yeast development (Figure 4E). However, the symptoms were not indicative of ‘aqueous spot’ evolution. 

Most mould species infected all inoculated wounds (100.0% of incidence) with symptoms as shows Figure 4F (*F. oxisporum*), Figure 4G (*P. crustosum*), Figure 4H (*A. alternata*), Figure 4I (*S. commune*), Figure 4J (*M. laxa*) and Figure 4K1,K4 (*B. cinerea*). The other inoculated mould species presented less infection capacity. *Mucor plumbeus* and *T. atroviride* presented 0.0% incidence, *C. cladosporioides* presented 36.7%, and *M. racemosus* and *A. infectoria* showed 56.7%. Interestingly, the infection evolution of the inoculated moulds demonstrated that *B. cinerea* infections (Figure 4K1) showed similar symptoms to cherries in the last stage of this disease (Figure 1F and Figure 3B), and other morphotypes less prevalent in the ‘aqueous spot’ were developed (Figure 4K2,K4). Neither of these moulds exhibited the ability to infect sound cherries by immersion in the conidia suspension. 

**Figure 5:**
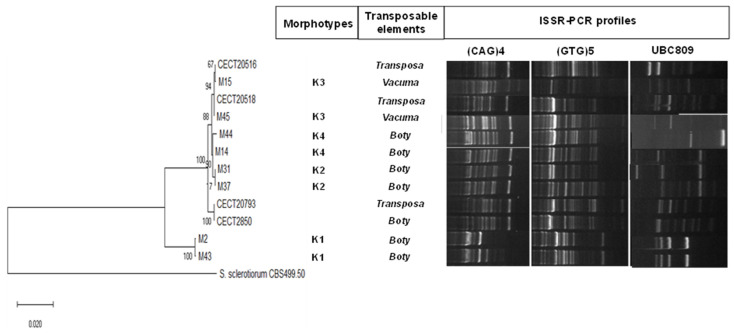
Analysis of the genetic variability of the four morphotypes utilising phylogenetic tree inferred by using the maximum likelihood method and Tamura–Nei model constructed with the concatenated sequences of ITS1-5.8S rDAN-ITS4 + LSU 26S rDNA+ calmodulin gene + β-tubulin of *Botrytis cinerea* morphotypes and type strains (the percentage of trees in which the associated taxa clustered together is shown next to the branches); detection of the transposable elements *Boty* and *Flipper* was registered as *Vacuma* (absence of transposable elements), *Boty* (presence of *Boty* element alone), and *Transposa* (presence of *Boty* and *Flipper* elements); and (**C**) ISSR profiles obtained with (CAG)_4_, (GTG)_5_, and UBC809.

Genetic characterisations of the different morphotypes of *B. cinerea* were carried out, selecting two representative isolates of each morphotype. Four reference strains were included in this work. The phylogenetic tree constructed with the concatenated sequences of ITS1-5.8S rDAN-ITS4 + LSU 26S rDNA+ calmodulin gene + β-tubulin is presented in Figure 5. The representative isolates of morphotype K1 (white mycelium) were clustered separately from the rest of the morphotypes and reference strains. The K1 sequences did not show differences in LSU 26S rDNA sequences with respect to the rest of *B. cinerea* strains, and only one single nucleotide polymorphism (substitution of A by T) was detected in ITS1-5.8S rDNA-ITS4. On the other hand, significant differences were found between white mycelium morphotypes and the rest in the other analysed genes, with percentages of identities 89.3% to 98.6% for calmodulin sequences and 94.1% to 94.3% for β-tubulin sequences. High genetic differences among K1 morphotypes and the rest of the strains were revealed by analysing ISSR-PCR profiles. Thus, the profiles with (CAG)_4_, (GTG)_5_, and UBC809 were unique and consistent in white mycelium *B. cinerea* strains.

The results of the double and triple inoculations are shown in Table 2. *Botrytis cinerea* M2 was selected as a base for this assay. The combination with bacteria better represented the ‘aqueous spot’ symptoms than *B. cinerea* M2 alone, with 100% of cherries infected. Among them, the combination with *L. quercina* and *L. mesenteroides* showed a higher degree of rotting because the browning area was larger.

Co-inoculation of *B. cinerea* M2 with yeast presented a disorder incidence between 26.7% (combined with *M. pulcherrima*) and 100% (*Y. lipolytica*, *C. railenensis*, *H. uvarum*, and *M. viticola*). Nevertheless, the degree of rotting was lower in all combinations, and mycelia development was delayed. 

Lastly, triple combinations were tested, using *L. quercina* and *T. terrea* as bacteria, and *Y. lipolytica*, *A. pullulans*, *H. uvarum*, and *M. viticola* based on their ubiquity and symptoms. All combinations developed symptoms associated with this disorder (100% incidence). However, the degree of rotting was lower in all cases than *B. cinerea* M2 alone. The areas of degraded tissues were smaller, and the appearance of visual mycelia was delayed.

## 4. Discussion

The emerging disease called ‘aqueous spot’ is a serious issue for cherry producers in the Jerte Valley (Cáceres, Spain). The initial symptoms begin before harvest but are often detected during the commercialisation of cherries. The events or agents that trigger this disease have remained unclear until now. A common feature in fruits deeply affected by ‘aqueous spot’ is the external development of a characteristic mycelium, which is primarily white and eventually covers the cherries completely. Mould counts in the early stages of ‘aqueous spot’ were beneath the detection thresholds, with inconsistent presence of various well-known rotting species. *Monilia laxa* [9], *A. alternata* [37], *P. crustosum* [11], *F. oxysporum* [38] and *B. cinerea* [13] have been associated with different diseases of sweet cherries. Inoculation trials showed a high disease incidence in most cases. White mycelial were observed in the wounded cherries inoculated with *F. oxysporum* (Figure 4F), *A. alternata* (Figure 4H), *S. commune* (Figure 4G), and *B. cinerea* (Figure 4K1). However, the evolution and morphology of white *B. cinerea* was uniquely compatible with ‘aqueous spot’ symptoms. The consistent isolation of white *B. cinerea* at the last stages of the disease confirmed its association with ‘aqueous spot’. Mycelium presence within the flesh of cherries at the early stages of ‘aqueous spot’ development, even in surface-sanitised fruits, placed this mould at the origin of symptoms. The mode of infection of this morphotype of *B. cinerea* remains unclear; however, growth failure with immersion inoculation demonstrated that wounds or latent infections [39] may be involved in its presence and development. The analysis of the levels of expression of some virulence genes during the infection could help to understand it. The fact that early cherry cultivars, such as ‘Burlat’, and a few late cultivars, such as ‘Lapins’ and ‘Sweetheart’, are prone to suffer ‘aqueous spot’ will need to be studied in future works. Previous studies indicated that cherry cultivars are susceptible to a wide range of pathogens [40], which may be influenced by canopy structure, crop load and blooming times, as well as the susceptibility to bounds, cracking, or mechanical damage due to genotypic differences [14,41].

In this work, *Botrytis* spp. isolates associated with ‘aqueous spot’ pathology displayed a wide morphological (Figure 4K) and genetic variability (Figure 5). Previous works have demonstrated the extensive phenotypic and genetic diversity of *B. cinerea* from different origins [35,42]; however, the reports of white mycelium *Botrytis* are not common. It has been postulated the division of *B. cinerea* into two sympatric species, *Vacuma* and *Transposa*, defined by the presence of the transposable elements *Boty* and *Flipper* [43], which are related to its pathogeny and resistance to fungicides. Morphotypes K1, K2, K4, and CECT 2850 were classified as *Boty*, whereas the K3 morphotype was classified as *Vacuma* (did not contain any transposable elements) (Figure 5). The strains of another type were classified as *Transposa*, which had both transposable elements. 

Bacteria and yeast populations were modified by the development of symptoms of this disease. Microbial counts (TAM and yeasts) of damaged cherries increased around one log_10_ CFU/g of cherries compared to sound tissues. In general, mean counts, even in sound tissues, were higher than those reported by Serradilla et al. [4]. Sanitising cherries with sodium hypochlorite reduced counts [44], but they were still higher than previously reported. This fact could be associated with increased spoilage of cherries in 2020. Wide variability in the microbial dominance among samples was detected, although the mean counts indicated that bacteria counts in damaged tissues were higher (around 0.5 log_10_ CFU/g, *p* < 0.050). Members of the order Enterobacterales dominated the bacterial population of damaged and sound tissues in all samples from both seasons. *Rahnella* spp., *Erwinia* spp. and *R. nectarea* have been reported as an epiphytic population of phyllosphere from different species of fruit trees [4,45,46]. The presence of *T. terrea* could be associated with vectors, such as *Drosophila suzukii* [47]. This group of bacteria are usually not related to fruit rot or spoilage, beyond a recent report on *P. agglomerans* [48]. Overall, the bacteria isolated from damaged cherries had a limited capacity for spoiling tissues around the wounds in individual inoculations. *Lonsdalea quercina* showed the highest occurrence (Figure 4A), producing fluid and browning of tissues around the wound. References have associated this species with canker disease in poplar trees [49]. Some bacteria, such as *Erwinia* spp., produced bubbles in the inoculated area, a symptom not associated with ‘aqueous spot’. The combination of bacteria and *B. cinerea* M2 showed similar incidence and symptoms to ‘aqueous spot’ than M2 inoculated alone. However, the combinations with *L. quercina* and *L. mesenterioides* showed more rotting tissues around wounds. Bacteria, by itself, showed low pathogenic capacity. Therefore, the results revealed that events that originate ‘aqueous spot’ symptoms favoured bacteria multiplication, and the symptoms could be enhanced.

The yeast population was increased by ‘aqueous spot’ events, as previously mentioned. The species identified commonly inhabit fruits’ surfaces. Specifically, *A. pullulans*, *H. uvarum*, *P. kluyveri* and *M. pulcherrima* have been identified in cherries [4,50]. Differences between yeast species were observed between seasons. *Y. lipolytica* was isolated in all samples in 2019, while *M. pulcherrima* and *M. viticola* appeared in all samples in 2020. Overall, the results of individual inoculations showed less incidence of yeasts, except for *G. candidum* (100% incidence), previously associated with sour rot in stone fruit [20]. The involvement of yeasts at the initial stages of ‘aqueous spot’ would be unlikely according to the results of the combined inoculations. The inhibition of *B. cinerea* M2 was detected in all combinations, decreasing the incidence in double inoculations, and delaying the appearance of visual mycelia in double and triple inoculations. The colonisation of wounds and competition for space and nutrients could explain this finding. Additionally, several strains of this yeast species have shown antagonistic activities, such as *M. pulcherrima*, *H. uvarum* [50,51,52], *C. oleophila* [53], and *A. pullulans* [54].

The present work describes for the first time the microbial population involved in the development of ‘aqueous spot’, an emerging disease of sweet cherries from the Jerte Valley. Genetic identifications and inoculation trials indicated that white mycelium *B. cinerea* were found at the early stages of this disease. This morphotype showed a wide genetic divergence with other *B. cinerea* strains, remarkably in calmodulin and β-tubulin gene sequences and ISSR-PCR profiles. Members of the order Enterobacterales showed a synergistic effect with *B. cinerea*, increasing the degree of rotting, while yeasts, at the initial stage, had an inhibitory effect on symptom development. Although this work demonstrates the involvement of white mycelium *B. cinerea* in this disease, further research using molecular biology techniques such as qRT-PCR or next-generation sequencing is needed to fully understand the origin and development of ‘aqueous spot’ in order to develop strategies for its control.

## Figures and Tables

**Table 1:** Microbial identification of bacteria, yeasts, and moulds isolated in 2019 and 2020 from cherries with ‘aqueous spot’ symptoms, and disease incidence of each species.

Season	Microbial Group	Species Identification	% of Identification (Reference Accession Number)	Disease Incidence (%)
2019	Bacteria	*Bacillus cereus*	100 (KF835392.1)	0.0
2019		*Agrococcus lahaulensis*	99.87 (MT214266.1)	0.0
2019, 2020		*Rahnella aquatilis*	100 (MN826597.1)	13.3
2019, 2020		*Tatumella terrea*	99.73 (LC505503.1)	43.3
2019, 2020		*Pantoea agglomerans*	99.73 (FJ611832.1)	20.0
2019, 2020		*Lonsdalea quercitina*	100 (JF311446.1)	83.3
2019, 2020		*Erwinia bilingiae*	100 (CP031695.1)	33.3
2019, 2020		*Erwinia persicina*	100 (MN540710.1)	13.3
2019, 2020		*Rosenbergiella nectarea*	100 (HQ284897.1)	36.6
2019, 2020		*Hafnia paralvei*	99.77 (MF111316.1)	13.3
2020		*Pseudomonas* spp.	100 (LC548103.1)	26.7
2020		*Leuconostoc mesenteroides*	100 (MT544938.1)	36.7
2019	Yeasts	*Yarrowia lipolytica*	100 (MH459414.1)	0.0
2019		*Rhodotorula nothofagi*	100 (KX811212.1)	0.0
2019, 2020		*Candida oleophila*	99.83 (MF940128.1)	0.0
2019, 2020		*Aureobasidium pullulans*	100 (MT035961.1)	0.0
2019, 2020		*Filobasidium wieringae*	100 (KY103450.1)	0.0
2019, 2020		*Filobasidium magnum*	100 (MH197140.1)	0.0
2019, 2020		*Candida railenensis*	99.52 (KY102357.1)	0.0
2019, 2020		*Pichia kluyveri*	99.54 (KY104557.1)	0.0
2019, 2020		*Hanseniaspora uvarum*	99.86 (KY103554.1)	13.3
2020		*Metschnikowia pulcherrima*	97.02 (CP034457.1)	20.0
			99.40 (CP034456.1) *	
2020		*Metschnikowia viticola*	92.66 (KY104213.1)	33.3
			100 (JN544019.1) *	
2020		*Geotrichum candidum*	99.80 (MN861070.1)	100
2019	Moulds	*Schizophyllum commune*	100 (MN218205.1)	100
2019		*Trichoderma atroviride*	100 (KU942400.1)	0.0
2019, 2020		*Penicillium crustosum*	100 (MK102704.1)	100
2019, 2020		*Mucor plumbeus*	99.67 (MK268150.1)	0.0
2019, 2020		*Alternaria alternata*	100 (MT446174.1)	100
2019, 2020		*Cladosporium cladosporioides*	100 (MF475952.1)	36.7
2019, 2020		*Botrytis cinerea*	100 (MH860108.1)	100
2020		*Fusarium oxysporum*	100 (MT529329.1)	100
2020		*Mucor racemosus*	99.84 (HM641690.1)	56.7
2020		*Monilia laxa*	100 (MN049483.1)	100
2020		*Alternaria infectoria*	100 (MH205934.1)	56.7

* D1/D2 domain of the 26S rRNA region.

**Table 2:** Effect of combined inoculations of bacteria, moulds, and yeasts isolated from cherries with ‘aqueous spot’.

Inoculation Combinations			Symptoms ^a^	Disease Incidence (%)
Mould	Bacteria	Yeasts		
*Botrytis cinerea* M2	*Erwinia bilingiae* B1	- ^b^	+ (gas production)	100
	*Lonsdalea quercina* B22	-	++	100
	*Leuconostoc mesenteroides* B24	-	++	100
	*Tatumella terrea* B27	-	+	100
	*Rosenbergiella nectarea* B45	-	+	100
	-	*Yarrowia lipolytica* L2	+/−	100
	-	*Aureobasidium pullulans* L12	+/−	70.0
	-	*Candida oleophila* L69	+/−	50.0
	-	*Candida railenensis* L193	+/−	100
	-	*Hanseniaspora uvarum* L92	+/−	100
	-	*Metschnikowia pulcherrima* L25	+/−	26.7
	-	*Metschnikowia viticola* L211	+/−	100
	-	*Pichia kluyveri* L223	+/−	46.7
	*Lonsdalea quercitina* B22	*Yarrowia lipolytica* L2	+/−	100
		*Aureobasidium pullulans* L12	+/−	100
		*Metschnikowia viticola* L211	+/−	100
		*Hanseniaspora uvarum* L92	+/−	100
	*Tatumella terrea* B27	*Yarrowia lipolytica* L2	+/−	100
		*Aureobasidium pullulans* L12	+/−	100
		*Metschnikowia viticola* L211	+/−	100
		*Hanseniaspora uvarum* L92	+/−	100

^a^ Development of ‘aqueous spot’ symptoms: browning of tissues and mycelium development. +: symptoms are more similar to ‘aqueous spot’ than *B. cinerea* M2 alone; +/−: decreased symptoms; ++: increased symptoms. ^b^ (-) microorganism that is not included in the inoculation combination.

## Data Availability

Data sharing does not apply to this article.

## Supplementary Materials

The following are available online at https://www.mdpi.com/article/10.3390/foods10102281/s1, Figure S1: Counts of total aerobic mesophilic and yeast in damaged and sound tissue from all sweet cheery cultivars after sanitisation.

## References

[1] Ministry of Agriculture, Fisheries and Food Home Page. https://www.mapa.gob.es/.

[2] Serradilla M.J., Martín A., Aranda E., Hernández A., Benito M.J., Lopez-Corrales M., Córdoba M.D.G. (2008). Authentication of “Cereza del Jerte” sweet cherry varieties by free zone capillary electrophoresis (FZCE). Food Chem..

[3] Serradilla M.J., Hernández A., Ruiz-Moyano S., Benito M.J., Corrales M.L., Cordoba M.D.G. (2012). Authentication of ‘Cereza del Jerte’ cherry cultivars using real time PCR. Food Control.

[4] Serradilla M.J., Villalobos M.D.C., Hernández A., Martín A., Lozano M., Córdoba M.D.G. (2013). Study of microbiological quality of controlled atmosphere packaged ‘Ambrunés’ sweet cherries and subsequent shelf-life. Int. J. Food Microbiol..

[5] Bai J., Plotto A., Spotts R., Rattanapanone N. (2011). Ethanol vapor and saprophytic yeast treatments reduce decay and maintain quality of intact and fresh-cut sweet cherries. Postharvest Biol. Technol..

[6] Wang Y., Xie X., Long L.E. (2014). The effect of postharvest calcium application in hydro-cooling water on tissue calcium content, biochemical changes, and quality attributes of sweet cherry fruit. Food Chem..

[7] Chen F., Liu X., Schnabel G. (2013). First report of brown rot caused by Monilinia fructicola in sweet cherry in maryland. Plant Dis..

[8] Liu Z., Bai H., Yang H., Tang S., Wei M., Huang X., Li Y. (2012). Biological characteristics of Monilia fructigena as pathogen of brown rot in sweet cherry. J. Fruit Sci..

[9] Kiprovski B., Borković B., Malenčić D., Veberic R., Štampar F., Mikulic-Petkovsek M. (2018). Postharvest changes in primary and secondary metabolites of sweet cherry cultivars induced by *Monilinia laxa*. Postharvest Biol. Technol..

[10] De Paiva E., Serradilla M.J., Ruiz-Moyano S., Cordoba M.D.G., Del Villalobos M., Casquete R., Hernández A. (2017). Combined effect of antagonistic yeast and modified atmosphere to control *Penicillium expansum* infection in sweet cherries cv. Ambrunés. Int. J. Food Microbiol..

[11] López S.N., Sangorrín M.P., Pildain M.B. (2016). Fruit rot of sweet cherries and raspberries caused by *Penicillium crustosum* and *Mucor piriformis* in South Patagonia, Argentina. Can. J. Plant Pathol..

[12] Wang C., Niu Y., Meng Q., Zhang L. (2019). Ethyl pyruvate (EP) suppressed post-harvest blue mold of sweet cherry fruit by inhibiting the growth of *Penicillium oxalicum*. J. Sci. Food Agric..

[13] Aktaruzzaman M., Afroz T., Kim B.-S., Lee Y.-G. (2016). Occurrence of postharvest gray mold rot of sweet cherry due to *Botrytis cinerea* in Korea. J. Plant Dis. Prot..

[14] Tarbath M., Measham P.F., Glen M., Barry K.M. (2014). Host factors related to fruit rot of sweet cherry (*Prunus avium* L.) caused by *Botrytis cinerea*. Australas. Plant Pathol..

[15] Zhao Y.Z., Liu Z.H. (2012). First report of black spot disease caused by *Alternaria alternata* on cherry fruits in China. Plant Dis..

[16] Romanazzi G., Nigro F., Ippolito A., Salerno M. (2001). Effect of short hypobaric treatments on postharvest rots of sweet cherries, strawberries and table grapes. Postharvest Biol. Technol..

[17] Ayyanath M.-M., Zurowski C.L., Scott I.M., Lowery D.T., Watson M.C., O’Gorman D.T., MacKenzie K.E., Úrbez-Torres J.R. (2018). Relationship between *Drosophila suzukiiand* postharvest disorders of sweet cherry (Prunus avium). Phytobiomes J..

[18] O’Gorman D.T., Walker M., Fraser J., Boulé J., Úrbez-Torres J.R., Toivonen P.M. (2016). Unravelling cherry slip-skin maceration disorder. Can. J. Plant Pathol..

[19] Kim Y.K. (2014). First report of a new postharvest rot in sweet cherries caused by *Aureobasidium pullulans*. Plant Dis..

[20] Michailides T.J., Morgan D.P., Day K.R. (2004). First report of sour rot of California peaches and nectarines caused by yeasts. Plant Dis..

[21] Alam M.W., Rehman A., Malik A.U., Iqbal Z., Amin M., Ali S., Hameed A., Sarfraz S. (2017). First report of *Geotrichum candidum* causing postharvest sour rot of peach in Punjab, Pakistan. Plant Dis..

[22] Ménard M., Sutra L., Luisetti J., Prunier J.P., Gardan L. (2003). Pseudomonas syringae pv. avii (pv. nov.), the causal agent of bacterial canker of wild cherries (*Prunus avium*) in France. Eur. J. Plant Pathol..

[23] Vicente J.G., Roberts S.J. (2007). Discrimination of Pseudomonas syringae isolates from sweet and wild cherry using rep-PCR. Eur. J. Plant Pathol..

[24] Spadoni A., Ippolito A., Sanzani S.M. (2020). First report of *Stemphylium eturmiunum* causing postharvest rot of sweet cherry in Italy. Crop. Prot..

[25] Serradilla M.J., Lozano M., Bernalte M.J., Ayuso M.C., Corrales M.L., González-Gómez D. (2011). Physicochemical and bioactive properties evolution during ripening of ‘Ambrunés’ sweet cherry cultivar. LWT.

[26] Ruiz-Moyano S., Totten S.M., Garrido D.A., Smilowitz J.T., German J.B., Lebrilla C.B., Mills D.A. (2013). Variation in consumption of human milk oligosaccharides by infant gut-associated strains of *Bifidobacterium breve*. Appl. Environ. Microbiol..

[27] Gallardo G., Ruiz-Moyano S., Hernández A., Benito M., Córdoba M., Pérez-Nevado F., Martín A. (2014). Application of ISSR-PCR for rapid strain typing of *Debaryomyces hansenii* isolated from dry-cured Iberian ham. Food Microbiol..

[28] White T.J., Bruns T., Lee S., Taylor J., Innis M.A., Gelfand D.H., Sninsky J.J., White T.J. (1990). Amplification and direct sequence of fungal ribosomal RNA genes for phylogenetics. PCR Protocols: A Guide to Methods and Applications.

[29] O’Donnell K., Reynolds R., Taylor J.W. (1993). Fusarium and its near relatives. The Fungal Holomorph: Mitotic, Meiotic and Pleomorphic Speciation in Fungal Systematic.

[30] Hong S.-B., Go S.-J., Shin H.-D., Frisvad J.C., Samson R.A. (2005). Polyphasic taxonomy of *Aspergillus fumigatusand* related species. Mycologia.

[31] Glass N.L., Donaldson G.C. (1995). Development of primer sets designed for use with the PCR to amplify conserved genes from filamentous ascomycetes. Appl. Environ. Microbiol..

[32] Tamura K., Nei M. (1993). Estimation of the number of nucleotide substitutions in the control region of mitochondrial DNA in humans and chimpanzees. Mol. Biol. Evol..

[33] Kumar S., Stecher G., Li M., Knyaz C., Tamura K., Battistuzzi F.U. (2018). MEGA X: Molecular evolutionary genetics analysis across computing platforms. Mol. Biol. Evol..

[34] Mahmoud M.A., Ali H.M., El-Aziz A.R.M., Al-Othman M.R., Al-Wadai A.S. (2014). Molecular characterization of aflatoxigenic and non-aflatoxigenic *Aspergillus flavus* isolates collected from corn grains. Genet. Mol. Res..

[35] Muñoz G., Hinrichsen P., Brygoo Y., Giraud T. (2002). Genetic characterisation of *Botrytis cinerea* populations in Chile. Mycol. Res..

[36] Sipiczki M., Pfliegler W.P., Holb I.J. (2013). *Metschnikowia* species share a pool of diverse rRNA genes differing in regions that determine hairpin-loop structures and evolve by reticulation. PLoS ONE.

[37] Ahmad T., Liu Y., Shujian H., Moosa A. (2020). First record of *Alternaria alternata* causing postharvest fruit rot of sweet cherry (*Prunus avium*) in China. Plant Dis..

[38] Úrbez-Torres J.R., Boulé J., Haag P., Hampson C., O’Gorman D.T. (2016). First report of root and crown rot caused by *Fusarium oxysporum* on sweet cherry (*Prunus avium*) in British Columbia. Plant Dis..

[39] Adaskaveg J.E., Förster H., Thompson D.F. (2000). Identification and etiology of visible quiescent infections of *Monilinia fructicola* and *Botrytis cinerea* in sweet cherry fruit. Plant Dis..

[40] Brown S.K., Wilcox W.F. (1989). Evaluation of cherry genotypes for resistance to fruit infection by *Monilinia fructicola* (Wint.) honey. HortScience.

[41] Param N., Zoffoli J.P. (2016). Genotypic differences in sweet cherries are associated with the susceptibility to mechanical damage. Sci. Hortic..

[42] Mirzaei S., Goltapeh E.M., Shams-Bakhsh M., Safaie N., Chaichi M. (2009). Genetic and phenotypic diversity among *Botrytis cinerea* Isolates in Iran. J. Phytopathol..

[43] Ma Z., Michailides T.J. (2005). Genetic structure of *Botrytis cinerea* populations from different host plants in California. Plant Dis..

[44] Zoffoli J.P., Toivonen P., Wang Y., Quero-García J., Lezzoni A., Puławska J., Lang G. (2017). Postharvest biology and handling for fresh markets. Cherries: Botany, Production and Uses.

[45] Halpern M., Fridman S., Atamna-Ismaeel N., Izhaki I. (2013). Rosenbergiella nectarea gen. nov., sp. nov., in the family Enterobacteriaceae, isolated from floral nectar. Int. J. Syst. Evol. Microbiol..

[46] Palacio-Bielsa A., Roselló M., Llop P., López M.M. (2011). Erwinia spp. from pome fruit trees: Similarities and differences among pathogenic and non-pathogenic species. Trees.

[47] Chandler J.A., James P.M., Jospin G., Lang J.M. (2014). The bacterial communities of *Drosophila suzukii* collected from undamaged cherries. PeerJ.

[48] Li C., Jia Y., Tian Y., Zhou L., Sun W., Deng J., Liu F. (2020). First report of necrotic disease caused by *Pantoea agglomerans* on plum (*Prunus salicina*) in China. Plant Dis..

[49] Tóth T., Lakatos T., Koltay A. (2013). *Lonsdalea quercina* subsp. *populi* subsp. *nov.*, isolated from bark canker of poplar trees. Int. J. Syst. Evol. Microbiol..

[50] Hamby K.A., Hernández A., Boundy-Mills K., Zalom F.G. (2012). Associations of yeasts with spotted-wing drosophila (*Drosophila suzukii*; *Diptera*: *Drosophilidae*) in cherries and raspberries. Appl. Environ. Microbiol..

[51] Ruiz-Moyano S., Martín A., Villalobos M., Calle A., Serradilla M., Cordoba M.D.G., Hernández A. (2016). Yeasts isolated from figs (*Ficus carica* L.) as biocontrol agents of postharvest fruit diseases. Food Microbiol..

[52] Ruiz-Moyano S., Hernández A., Galvan A.I., Córdoba M.G., Casquete R., Serradilla M.J., Martín A. (2020). Selection and application of antifungal VOCs-producing yeasts as biocontrol agents of grey mould in fruits. Food Microbiol..

[53] Saligkarias I., Gravanis F., Epton H.A. (2002). Biological control of *Botrytis cinerea* on tomato plants by the use of epiphytic yeasts *Candida guilliermondii* strains 101 and US 7 and *Candida oleophila* strain I-182: II. A study on mode of action. Biol. Control..

[54] Mari M., Martini C., Spadoni A., Rouissi W., Bertolini P. (2012). Biocontrol of apple postharvest decay by *Aureobasidium pullulans*. Postharvest Biol. Technol..

