# Supplementary material for: Identification of the Causal Agent of Aqueous Spot Disease of Sweet Cherries (Prunus avium L.) from the Jerte Valley (Cáceres, Spain)
Source: Foods. 2021 Sep 26;10(10):2281. doi: 10.3390/foods10102281 (PMC8534920; doi:10.3390/foods10102281)
Supplement: Supplementary file 1 [file foods-10-02281-s001.zip › foods-1348676-SI.pdf]

foods

MDPI

Identification of the causal agent of aqueous spot disease of sweet cherries (*Prunus avium* L.) from the Jerte Valley (Cáceres, Spain)

Manuel J. Serradilla[1,a], Carlos Moraga,[2,3,a], Santiago Ruiz-Moyano[2,3*], Paula Tejero[2,3], María G. Córdoba[2,3,] Alberto Martín[2,3], Alejandro Hernández[2,3]

SUPPLEMENTAL MATERIAL

This article contains a supplementary Figure

Figure S1

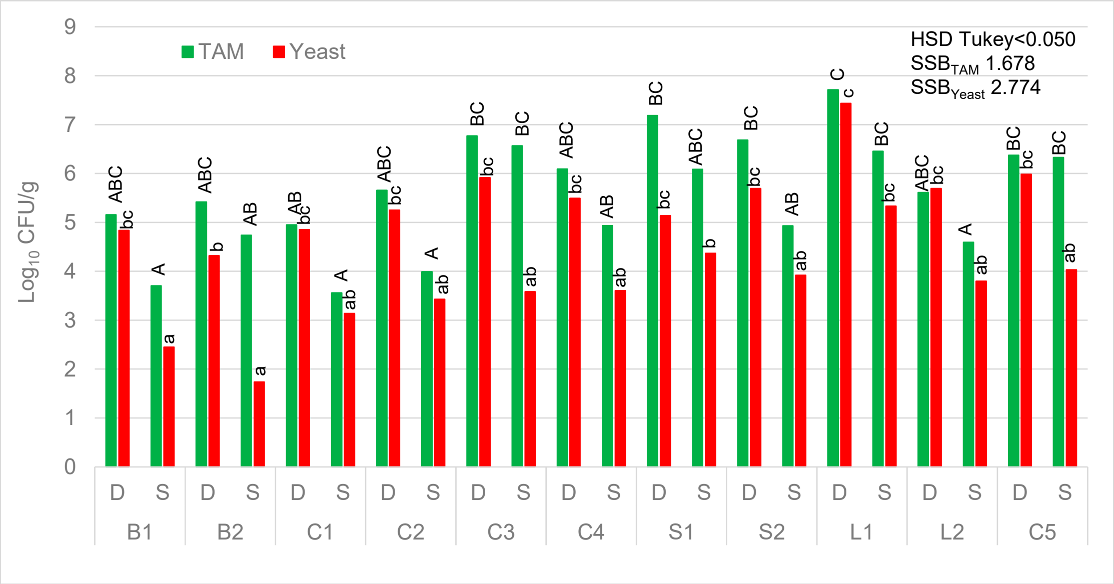

Figure S1. Counts of total aerobic mesophilic (TAM) microorganisms and yeast in damaged (D) and sound tissue (S) from (A) samples of cherries in 2019 (B1, B2, C1-C4, S1, S2) and 2020 (L1, L2, C5) after sanitisation with sodium hypochlorite. Bars with different upper-case letters for TAM and lower-case letter for yeast indicates statistical differences ($p < 0.050$). B, C, S, and L represents 'Burlat', 'California', 'Sweetheart', and 'Lapins' sweet cherry cultivars, respectively. HSD Tukey: Honestly Significant Difference; SSB: Statistical Significance Bar.
